# Indicators of dietary patterns in Danish infants at 9 months of age

**DOI:** 10.3402/fnr.v59.27665

**Published:** 2015-06-24

**Authors:** Louise B.B. Andersen, Christian Mølgaard, Kim F. Michaelsen, Emma M. Carlsen, Rasmus Bro, Christian B. Pipper

**Affiliations:** 1 Department of Nutrition, Exercise and Sports, Faculty of Sciences, University of Copenhagen, Copenhagen N, Denmark; 2 Department of Pediatrics, Copenhagen University Hospital, Hvidovre, Denmark; 3 Department of Food Science, University of Copenhagen, Frederiksberg, Denmark; 4 Department of Public Health, University of Copenhagen, Copenhagen K, Denmark

**Keywords:** SKOT, dietary patterns, family, maternal, paternal, child, characteristics

## Abstract

**Background:**

It is important to increase the awareness of indicators associated with adverse infant dietary patterns to be able to prevent or to improve dietary patterns early on.

**Objective:**

The aim of this study was to investigate the association between a wide range of possible family and child indicators and adherence to dietary patterns for infants aged 9 months.

**Design:**

The two dietary patterns ‘Family Food’ and ‘Health-Conscious Food’ were displayed by principal component analysis, and associations with possible indicators were analysed by multiple linear regressions in a pooled sample (*n*=374) of two comparable observational cohorts, SKOT I and SKOT II. These cohorts comprised infants with mainly non-obese mothers versus infants with obese mothers, respectively.

**Results:**

A lower Family Food score indicates a higher intake of liquid baby food, as this pattern shows transition from baby food towards the family's food. Infants, who were younger at diet registration and had higher body mass index (BMI) *z*-scores at 9 months, had lower Family Food pattern scores. A lower Family Food pattern score was also observed for infants with immigrant/descendant parents, parents who shared cooking responsibilities and fathers in the labour market compared to being a student, A lower Health-Conscious Food pattern score indicates a less healthy diet. A lower infant Health-Conscious Food pattern score was associated with a higher maternal BMI, a greater number of children in the household, a higher BMI *z*-score at 9 months, and a higher infant age at diet registration.

**Conclusions:**

Associations between infant dietary patterns and maternal, paternal, household, and child characteristics were identified. This may improve the possibility of identifying infants with an increased risk of developing unfavourable dietary patterns and potentially enable an early targeted preventive support.

Diet during infancy is important for health, both early and later in life, and tracking of eating habits into later childhood and adulthood has been observed (1–3). When the infant's diet gradually change from breast milk or infant formula to the family's diet during the complementary feeding period, the complexity of the diet increases. In research, the dietary intake has traditionally been characterised by one or a few nutrients. However, infants do not eat nutrients, they consume foods and meals; therefore, a characterisation based on a few nutrients might not be the most appropriate. The investigation of dietary patterns representing the whole diet rather than single nutrients might capture more of the diet's complexity and thereby increase our understanding of the relation between infant diet and health (4, 5). To be able to prevent or to improve undesirable dietary patterns in infancy, it is important to increase our awareness of indicators that are associated with different dietary patterns.

As far as we are aware, indicators of dietary patterns in children aged 0–36 months have been investigated in 12 papers based on seven studies (6–17). The indicators can roughly be divided into maternal, paternal, household, and child characteristics. These studies indicate that children with siblings (7–11, 13–15, 17), with low-educated mothers (6–11, 13–17), with young mothers (6, 7, 9–11, 13–17), and with mothers who have high body mass indexes (BMIs) (7–9, 13–16) have higher scores in unhealthy dietary patterns. However, little is known about these associations in infants who are younger than 12 months of age. Other possible indicators such as sex, body size, activity level of the child, person responsible for the cooking at home, and immigrant status are less investigated, and, to our knowledge, the possible paternal indicators are nearly unexplored (9, 10). Therefore, in our study, we aimed to investigate the association between a wide range of possible indicators and adherence to dietary patterns in infants aged 9 months when the complementary feeding period is well under way.

## Subjects and methods

### Study design and participants

Data were collected when infants were 9 months of age in the two observational cohorts, SKOT I and SKOT II, which had similar data-collection designs. In this paper, the data from the two cohorts are pooled, which is possible because of the similar designs. In SKOT I, the participants were infants randomly selected from the Danish capital area based on extractions from the National Civil Registration System and with no restrictions on maternal BMI. Inclusion criteria for participation in SKOT I required participants to be healthy singletons, born at 37–43 weeks of gestation, aged 9 months ±2 weeks at the first examination, and having Danish-speaking parents. Inclusion criteria for SKOT II were equal to SKOT I; in addition, all participants were required to be offspring of women with pre-pregnancy BMIs above 30 kg/m^2^ and who had participated in the intervention study, ‘Treatment of Obese Pregnant Women’ (TOP) at Hvidovre Hospital in the capital of Denmark (18, 19). Data were collected when infants were aged 9 months, for SKOT I in the year 2007–2008, and in the year 2011–2012 for SKOT II. The studies were conducted according to guidelines laid down in the declaration of Helsinki, and all procedures involving human subjects were approved by The Committees on Biomedical Research Ethics for the Capital Region of Denmark (SKOT I: H-KF-2007-0003 and SKOT II: H-3-2010-122). Written informed consents were obtained from all parents. Further details about study design and the suitability of pooling the two cohorts have been reported previously (20).

### Dietary data and dietary patterns

Using a validated 7-day food record method (21), the dietary intake of the infants was recorded by parents for seven consecutive days. Portion sizes were estimated with household measures and a photo booklet and noted in a pre-coded food diary. Intake of food items was calculated for each participant using the software General Intake Estimation System (GIES, version 1.000d, developed at the National Food Institute, Technical University of Denmark). In this study, the dietary intake was divided into 23 food groups (*Porridge, BreakfastCereals, WheatBreadWholegrain, WheatBreadNoWholegrain, RyeBread, PastaRice, Potato, Fruit, Vegetable, Fish, Meat, Poultry, Egg, FatsAnimal, FatsVegetable, Cheese, Milk, Formula, BreastMilk, FruitNutSnack, SweetsCake, SugaryDrink*, and *FastFood*) that encompassed the whole diet. These food groups represented intake in g/kg body weight/day, except *BreastMilk*, which represented feedings/day. Food groups were named with a short, compressed description, such as ‘*FatsAnimal*’ and ‘*SugaryDrink*’. The division of food items into groups, based on nutritional knowledge, was an attempt to cover most aspects of the official recommendations, the typical infant diet in Denmark and issues addressed in scientific studies on infant diet. A principal component analysis (PCA) was displayed with these food groups to identify latent dietary patterns, with the purpose of reducing the number of dietary variables, but reserving the information of the whole dietary intake representing underlying dietary concepts. Further details of these food groups and PCA have been reported previously (20).

### Possible indicators

The selection of possible indicators for dietary patterns in infants aged 9 months was based on published literature within this area (6–11, 13–15). Data regarding parental and household factors were collected when the infants were aged 9 months. Weights and heights of the fathers in both cohorts and of mothers in SKOT I were self-reported, while for the mothers in SKOT II, they were measured during the 9 months examination. BMI was calculated as weight (kg)/height (m)^2^. Parental immigrant/descendant status was recorded beginning with the infants’ grandparents. Total household income was divided into more or less than 650,000DKK (~US$120,000) per year. Average income for families in Denmark is 684,000DKK (~US$127,000).

Duration of breastfeeding, age at introduction of complementary feeding, and age of crawling were recalled by an interview and questionnaire at 9 months. Exclusive breastfeeding was defined as receiving only breast milk, water, and vitamins from birth. A question asking for the age at which the child first ate different kinds of typical complementary foods was used to estimate the age at introduction of complementary feeding (0–3, 4, 5, 6, 7, and 8 months). Relative physical activity level was estimated by the parents to be more or less active as that of the infant's peers. Birth weight and length were obtained from health records kept by parents, with measurements carried out by midwives. At 9 months, weight and length were measured at the department of Nutrition, Exercise and Sports, University of Copenhagen, by trained research staff. Recumbent length was a mean of three measurements carried out with a digital measuring board (Force Technology, Brøndby, Denmark) which made recordings to the nearest 0.01 cm. Using a digital scale (Sartorius IP 65; Sartorius AG, Göttingen, Germany), weight was measured, without clothes, to the nearest 0.1 kg. BMIs (kg/m^2^) were converted to *z*-scores using the software program WHO Anthro 2005 and the WHO growth standards as a reference (22). The parents were requested to start the dietary assessment as close as possible to the 9 months visit.

### Statistical analyses

The indicator analyses were based on complete cases. Infants with missing values are referred to as ‘non-completers’. Categorical variables are represented as %, and completers were compared with non-completers via Pearson's chi-square test. Continuous variables are represented as mean and SD, and completers were compared with non-completers via the unpaired *t*-test (for equal variance) or Welsh's *t*-test (for unequal variance) if normally distributed, and represented as median, 25;75 percentiles and compared via Mann–Whitney test if not. The numbers of children and adults in the households were descriptively presented as categorical variables but treated as continuous variables in the statistical tests.

Dietary patterns were identified by PCA, which was carried out in MATLAB R2010b using PLS_Toolbox Version 7.3.1, and included all participants with diet records; both complete and non-complete indicator cases. Data were autoscaled before the PCA, and the number of principal components was selected based on a clear change in the scree plot (23) and interpretability of the principal components. Dietary patterns were tentatively named based on subjective assessments of food groups with the highest loadings within each principal component to ease communication. Individual score values from each identified dietary pattern were used as continuous variables.

A multivariate linear regression model was fitted for each of the dietary patterns identified as the outcome variable and simultaneously included all the possible indicators as explanatory variables. The initial model for each dietary pattern included maternal work situation, maternal education level, maternal age at child's birth, maternal BMI when the infant was 9 months, paternal work situation, paternal education level, paternal age at child's birth, paternal BMI when the infant was aged 9 months, number of adults in the household, number of children in the household, household income, smoking in the home, parental immigrant/descendant status, person responsible for cooking at home, infant BMI *z*-score at birth, infant age at diet registration, infant BMI *z*-score at 9 months, duration of exclusive breastfeeding, infant age at the introduction of complementary feeding, whether the infant was crawling at 9 months, infant physical activity level at 9 months, infant in day care or home care at 9 months, and sex of the infant. Estimates from the reduced model after backward stepwise selection, with *p*<0.05 as the cut-off, are reported.

When reporting the reduced multivariate models, standardised regression coefficients were obtained by refitting the models after standardising both the outcomes and explanatory variables. For each variable, standardisation consists of subtraction of the sample mean and dividing by the sample standard deviation. Categorical explanatory variables were broken down into dummy variables before standardisation. Consequently, the standardised regression coefficient refers to how many standard deviations an outcome variable will change, per standard deviation increase in the explanatory variable. Univariate linear regressions with each of the dietary patterns and one indicator at a time were also conducted for comparison. All statistical tests were conducted in the statistical programming environment R version 3.0.2 (www.r-project.org). All *p*-values were evaluated at a 5% significance level.

## Results

### Characterisation of participants and dietary patterns

**Fig. 1:**
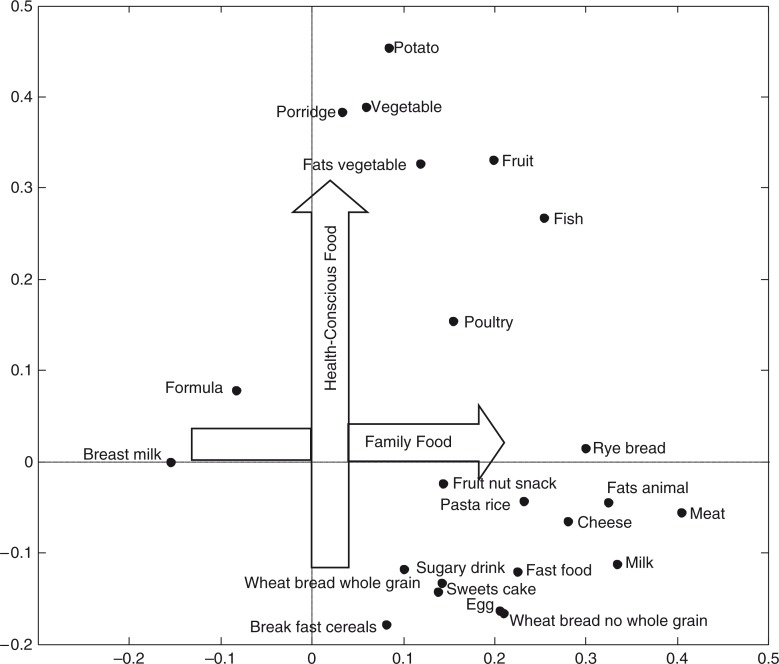
The two dietary patterns; Family Food and Health-Conscious Food at 9 months of age. Loading plot based on a PCA with intake of foods in g/kg body weight per day (except *BreastMilk*, which is in feedings per day) in a pooled sample of infants from the two cohorts SKOT I and SKOT II. Foods close to each other are positively correlated and foods placed in each end of a dietary pattern are inversely correlated. The Family Food pattern explained 13% and the Health-Conscious Food pattern explained 9% of the variation in dietary intake.

Two dietary patterns were identified, and they explain 13 and 9% of the variation in the intake of the food groups, respectively (Fig. 1). The first pattern was named Family Food and is an overall expression of the transition from *BreastMilk* and *Formula* to other foods because *BreastMilk* and *Formula* had the lowest loadings for this pattern, while foods such as *Meat*, *FatsAnimal*, *RyeBread*, and *Milk* had the highest loadings. The second pattern was named Health-Conscious Food because foods such as *SweetsCake* and *SugaryDrink* had the lowest loadings and *Potato*, *FatsVegetable*, *Fruit*, and *Vegetable* had some of the highest loadings in this pattern. Numerical values of the loadings are presented in the supplementary file.

The group of participants is characterised by including infants from very varied backgrounds in relation to parental socioeconomic status, parental obesity status, and breastfeeding history (Table 1). The infants included in the regression analyses (*n*=374) in this paper all had complete data sets. These completers differed from non-completers in a number of characteristics, for example, a higher proportion of completers came from SKOT I, had more highly educated parents, a lower maternal BMI, and less immigrant/descendant parents than non-completers.

**Table 1:** Characteristics of infants at 9 months, comparison of completers and non-completers

		Completers (*n*=374)		Non-completers (*n*=139)		
				
		% or mean or median	SD or 25;75 percentile	% or mean or median	SD or 25;75 percentile	*p*^a^
Cohort origin	SKOT I, %	74		37		
	SKOT II, %	26		63		**<0.001**^b^
Dietary patterns						
Family Food	Scores, mean, SD	0.032	1.699	−0.162	1.717	0.37^c^
Health-Conscious Food	Scores, mean, SD	0.12	1.43	−0.64	1.03	**<0.001**^d^
Maternal characteristics						
Work situation	Has job, %	79		74		
	Student, %	11		11		
	No job, %	10		15		0.36^b^
Education level^e^	Basic education, %	21		34		
	Short education, %	11		12		
	Medium education, %	33		32		
	Long education, %	35		22		**0.010**^b^
Age at child's birth	Years, mean, SD	31.9	4.4	31.2	5.5	0.29^d^
BMI when child is 9 months^f^	kg/m^2^, median, 25;75 percentiles	23.7	21.1;30.1	34.5	27.8;37.2	**<0.001**^g^
Paternal characteristics						
Work situation	Has job, %	88		88		
	Student, %	9		6		
	No job, %	3		6		0.33^h^
Education level	Basic education, %	31		48		
	Short education, %	9		9		
	Medium education, %	22		23		
	Long education, %	39		20		**0.003**^b^
Age at child's birth	Years, mean, SD	33.9	5.3	32.8	4.9	0.098^c^
BMI when child is 9 months	kg/m^2^, median, 25;75 percentiles	25.3	23.6;28.3	25.8	23.3;29.9	0.68^**g**^
Household characteristics						
No. of adults in household	1, %	2		15		
	2, %	98		83		
	>2, %	1		2		**<0.001**^g^
No. of children in household	1, %	57		59		
	2, %	33		30		
	3, %	7		10		
	>3, %	3		1		0.72^g^
Household income^i^	<650,000DKK, %	40		60		
	>650,000DKK, %	60		40		**0.001**^b^
Smoking in the home	Yes, %	98		90		
	No, %	2		10		**0.005**^h^
Parental immigrant/	Danish mother and father, %	80		68		
descendant status	Mother and/or father not Danish^j^, %	20		32		**0.009**^b^
Person who cooks at home	Mother/woman, %	57		64		
	Father/man, %	17		12		
	By turn or jointly, %	26		24		0.25^b^
Child characteristics						
BMI at birth	*z*-score, mean, SD	−0.26	1.00	−0.30	1.19	0.72^d^
Age at diet registration	months, mean, SD	8.9	0.4	9.0	0.6	**0.013**^d^
BMI at 9 months	*z*-score, mean, SD	0.42	1.0	0.35	0.88	0.50^c^
Duration exclusive BF	days, median, 25;75 percentiles	122	57;152	91	14;141	**0.022**^g^
Age at introduction of	0–3 months, %	5		11		
complementary feeding	4 months, %	60		58		
	5 months, %	26		20		
	6–7 months, %	9		12		0.069^b^
Crawling at 9 months	Yes, %	49		63		
	No, %	51		37		**0.047**^b^
Physical activity level at 9 months	More than others at same age, %	32		45		
	Same level as others at same age, %	62		53		
	Less than others at same age, %	6		3		0.083^h^
Day care or home care	Home care, %	94		79		
at 9 months	Day care, %	6		21		**<0.001**^b^
Sex	Girls, %	49		53		
	Boys, %	51		47		0.49^b^

BF, Breastfeeding.

a Pooled group of children from the SKOT I and SKOT II cohorts, in which completers (with complete data for diet and indicators used in regressions, Tables 2 and 3) are compared with non-completers (with at least one missing piece of data for diet or indicators).

b Pearson's chi-squared test.

c Unpaired *t*-test.

d Unpaired Welsh's *t*-test.

e Basic: basic/secondary school or preparatory education, Short: short-cycle higher education below 3 years, Medium: medium-cycle higher education 3-4 years, Long: long-cycle higher education above 4 years.

f SKOT I, self-reported in questionnaire; SKOT II, measured during the 9 months examination.

g Mann–Whitney test.

h Fisher's exact test.

i Country average for families is 684,000DKK (~US$127,000).

j 14% were from Scandinavia, 43% were from other European countries, and 42% were from outside Europe. P-values<0.05 is indicated with bold numbers.

### Indicators of the dietary pattern Family Food

The initial regression model included all the maternal, paternal, household, and child characteristics shown in Table 1 as possible indicators for the Family Food pattern. Both the full and the reduced model (Table 2) showed that a lower infant age at diet registration and higher BMI *z*-scores at 9 months were associated with lower scores in the Family Food pattern. Moreover, infants with immigrant/descendant parents, parents who share the cooking responsibilities, and fathers with jobs had lower scores in the Family Food pattern compared to infants of Danish parents, infants from families where the mother is the only home cook and infants with fathers who are students. Based on the confidence interval, including zero, no differences were seen between infants with a father without a job versus a father who has a job or between mothers versus fathers as the only home cook. Univariate regressions, including the Family Food pattern as the outcome variable and one of the possible indicators, by turn, as the explanatory variable, showed comparable results. Based on the standardised coefficients, it is seen that BMI *z*-scores at 9 months are the strongest indicators for the Family Food pattern followed by the infant's age at diet registration. There was a trend in the reduced model, in which a greater number of children in the household (*p*=0.08) were associated with higher scores in the Family Food pattern, but this was excluded from the reduced model presented. The variability accounted for in the reduced model was 12%.

**Table 2:** Indicators associated with the dietary pattern Family Food at 9 months of age (*n*=374) including standardised coefficients^a^

		Reduced multivariate regression model after stepwise backward selection^b^			
		
Indicators	Comparison of subgroups	*β*	95% CI	Std. *β*^c^	*p*
Infant age at diet registration (months)		0.90	0.52, 1.30	0.22	**<0.001**
Infant BMI 9 months (*z*-score)		−0.39	−0.56,−0.23	−0.23	**<0.001**
Paternal work situation					**0.004**^d^
	Student vs. has job *(ref)*	0.93	0.35, 1.5	0.15	
	No job vs. has job *(ref)*	0.61	−0.32, 1.5	0.063	
Parental immigrant/descendant status					**0.017**
	Mother and/or father not Danish vs. Danish mother and father *(ref)*	−0.50	−0.91, −0.089	−0.12	
Person who cooks at home					**0.016**^d^
	Father/man vs. Mother/woman *(ref)*	0.23	−0.22, 0.68	0.051	
	By turn or jointly vs. Mother/woman *(ref)*	−0.45	−0.84, −0.071	−0.12	
					Adj. *R*^2^=0.12

a Based on multiple linear regression with the initial full model, including maternal work situation, maternal education level, maternal age at child's birth, maternal BMI when the infant was 9 months, paternal work situation, paternal education level, paternal age at child's birth, paternal BMI when the infant was 9 months, number of adults in household, number of children in household, household income, smoking in the home, parental immigrant/descendant status, person who cooks at home, infant BMI *z*-score at birth, infant age at diet registration, infant BMI *z*-score at 9 months, duration of exclusive breastfeeding, infant age at introduction of complementary feeding, if the infant was crawling at 9 months, infant physical activity level at 9 months, infant in day care or home care at 9 months, and sex of the infant.

b Model reduction by backward stepwise selection until all *p*<0.05 in the model.

c Standardised regression coefficient; meaning how many standard deviations an outcome variable will change, per standard deviation increase in the explanatory variable.

d *p*-Value based on the collected variables before broken down to dummy variables. P-values<0.05 is indicated with bold numbers.

### Indicators of the dietary pattern Health-Conscious Food

The initial regression model included all the maternal, paternal, household, and child characteristics shown in Table 1 as possible indicators for the Health-Conscious Food pattern. Both the full and reduced regression models (Table 3) showed that higher maternal BMIs, greater numbers of children in the household, higher ages of the infant at diet registration, and higher BMI *z*-scores of the infants at 9 months were associated with lower scores in the Health-Conscious Food pattern. Univariate regressions, including the Health-Conscious Food pattern as the outcome variable and one of the possible indicators, by turn, as the explanatory variable, showed comparable results. Based on the standardised coefficients, it is seen that maternal BMI was the strongest indicator for the Health-Conscious Food pattern, followed by the age of the infant at diet registration. With 1 SD increase in maternal BMI, the score value of the Health-Conscious Food pattern decreased with 0.22 SD. The variability accounted for in the reduced model was 13%.

**Table 3:** Indicators associated with the dietary pattern Health-Conscious Food at 9 months of age (*n*=374) including standardised coefficients^a^

	Reduced multivariate regression model after stepwise backward selection^b^			
	
Indicators	*β*	95% CI	Std. *β*^c^	*p*
Infant age at diet registration (months)	−0.52	−0.88, −0.17	−0.15	**0.004**
Infant BMI at 9 months (*z*-score)	−0.16	−0.30, −0.023	−0.11	***0.022***
Maternal BMI when child is 9 months (kg/m^2^)	−0.051	−0.075, −0.027	−0.22	**<0.001**
Number of children in household	−0.20	−0.36, −0.037	−0.12	**0.017**
				Adj. *R*^2^=0.13

a Based on multiple linear regression with the initial full model, including maternal work situation, maternal education level, maternal age at child's birth, maternal BMI when the infant was 9 months, paternal work situation, paternal education level, paternal age at child's birth, paternal BMI when the infant was 9 months, number of adults in household, number of children in household, household income, smoking in the home, parental immigrant/descendant status, person who cooks at home, infant BMI *z*-score at birth, infant age at diet registration, infant BMI *z*-score at 9 months, duration of exclusive breastfeeding, infant age at introduction of complementary feeding, if the infant was crawling at 9 months, infant physical activity level at 9 months, infant in day care or home care at 9 months, and sex of the infant.

b Model reduction by backward stepwise selection until all *p*<0.05 in the model.

c Standardised regression coefficient; meaning how many standard deviations an outcome variable will change, per standard deviation increase in the explanatory variable. P-values<0.05 is indicated with bold numbers.

## Discussion

The strongest indicator for a lower Family Food score was a higher BMI *z*-score of the infant at 9 months, while a higher maternal BMI was the strongest indicator for a lower Health-Conscious Food score. Pooling of the two cohorts SKOT I and SKOT II increased the heterogeneity in the sample of infants, especially in relation to breastfeeding history, parental socioeconomic status, and obviously maternal obesity status, and thereby served as an interesting basis for investigating a wide range of possible indicators for the dietary patterns at 9 months of age. The observed associations suggest the relevance of investigating indicators for dietary patterns as early as 9 months of age.

### Indicators of the dietary pattern Family Food

Only two studies that we know of, the Southampton Women's Survey (13) and the Avon Longitudinal Study of Parents and Children (ALSPAC) (14), have investigated dietary patterns with PCA within the first year of life, both at 6 months of age. These two studies report dietary patterns specifically related to the complementary feeding period as we do, but their patterns are not directly equal to our Family Food pattern because of different methodologies, ages, and populations. However, our study does confirm findings from these two studies. We show that, at this early stage, dietary patterns are associated with characteristics of the infant as well as with characteristics of the infant's family. At 9 months, contrary to 6 months of age, the infant should be well under way in the complementary feeding transition, and it is thereby an important age on which to focus, though, at this age, the most desirable score value in the Family Food pattern is less obvious. Nevertheless, the Family Food pattern is useful for exploring indicators associated with the complementary feeding practice; however, confirmatory analyses, for example, by investigating the relation to nutrient intake, are needed to endorse health implications. This should be kept in mind when interpreting the direction of associations with indicators.

A higher rate of lifestyle-related diseases in immigrants compared to non-immigrants has previously been observed in Denmark (24), but the knowledge about dietary intake in early life among immigrants is sporadic (25). Thus, it is interesting to observe that infants with immigrant/descendant parents had significantly lower scores in the Family Food pattern than infants whose parents were both Danish. However, it should be stressed that it is unknown if our results indicate that the infants with immigrant/descendant parents are too far behind in the complementary feeding process towards family food at this age in a way that poses negative health consequences. The relatively high positive loading for *RyeBread*, which is a traditional Danish food, at the Family Food pattern might imply that this gradient especially reflects the Danish traditions of complementary feeding. Nevertheless, a previous questionnaire survey for Danish health visitors showed that infants of immigrant parents more often had an unbalanced diet and a late introduction of complementary foods (25). These findings might therefore be an enticement to continue research within this area. In the ALSPAC cohort, an association between the dietary patterns, specifically related to the complementary feeding period, and ethnicity was not found. However, it is interesting to note that, in the British ALSPAC cohort, infants and toddlers of non-white parents, compared to offspring of white parents, had higher scores in a healthy pattern (11, 14, 17). This contradicts our finding of no association between immigrant/descendant status and the Health-Conscious Food pattern. The authors at ALSPAC suggest that the association is influenced by the ethnicities included and the degree of adherence to the original food culture.

As a measure of socioeconomic status, paternal work situation was associated with the Family Food pattern. It indicated that infants with a father in the labour market ate more baby food and were less advanced in the transition towards family food compared to families in which the father was a student. No association was seen for fathers without jobs, but only few infants with complete data had fathers without jobs, which made a significant association less likely. We do not know of other studies investigating dietary patterns in early life with respect to the paternal work situation. However, 2-year-old Norwegian toddlers ate less baby food if the mother worked full-time than if the mother did not have a job (10). Moreover, we are not aware of other studies investigating the association between cooking responsibility in infant families and a Family Food like pattern, but one study has previously reported a healthier dietary pattern in 3-year-old children if the mother was the primary cook compared to another person (11). We did not, though, find any association between the Health-Conscious Food pattern and the person who cooked at home.

It could be hypothesised that the largest 9-month-old infants would be ahead in the complementary feeding period, as reported previously (26, 27), perhaps because they had an earlier need (or parents presumed they had an earlier need) for food other than breast milk or infant formula. Therefore, a positive association between BMI *z*-scores at 9 months and the Family Food pattern, instead of the inverse association we observed, would be expected.

### Indicators of the dietary pattern Health-Conscious Food

The association between higher maternal BMI and lower scores in the Health-Conscious Food pattern confirms previous findings (8, 13–16). This association appeared although a higher proportion of SKOT II infants compared to those in SKOT I were excluded because of non-complete data. Even though our analysis showed this association, it does not disclose whether maternal weight loss will result in healthier dietary patterns of the offspring. However, this might be possible because maternal and offspring dietary patterns are correlated (13), and maternal weight loss reduces offspring obesity risk (28). In contrast, no association was observed between the Health-Conscious Food pattern and paternal BMI, which is in agreement with a study of 14-month-old toddlers (9). Unfortunately, this is the only other study we know of reporting an investigation of the association between paternal BMI and dietary patterns in early life. However, it could be speculated that such an association will appear later in childhood, when maternity leave and breastfeeding is finished, making the parents more equal with respect to the child-feeding task. The association between the presence of siblings in the household and lower scores at a healthy dietary pattern has been repeatedly observed (8, 11, 13, 15). As suggested by North (11), older siblings might introduce less healthy food to the infant. The trend of higher scores in the Family Food pattern with higher numbers of children in the household also suggests that the complementary feeding period might be more rapid for infants with older siblings.

The association between age at diet registration and the Health-Conscious Food pattern probably indicates that this pattern, in addition to the Family Food pattern, also holds some information about how far the infant is in the complementary feeding process. *Potato*, *Vegetable*, *Fruit*, and *FatsVegetable*, which are used in mash, plus *Porridge* and *Formula*, are all typical complementary foods that have high loadings in the Health-Conscious Food pattern. An association between infant age at diet registration and healthy/unhealthy dietary patterns was also observed in two other studies with 14-month-old toddlers (6, 9), but not at a later examination at 24 months (6), which indicates that this association is transient and might fade when children have finished the complementary feeding period.

As suggested for the Family Food pattern, it could be hypothesised that parents feed their children differently depending on whether the child is small or big for its age. This might be supported by the association between a higher BMI *z*-score at 9 months and a lower score in the Health-Conscious Food pattern, indicating that infants with the largest body sizes eat less healthfully. However, the reverse causality, that infant body size depends on the diet, is perhaps more intuitive for this pattern. Two other studies with which we are familiar investigated current body size as a predictor of dietary patterns at 14 months (9) and 36 months (8). Contrary to our results, these studies with toddlers did not find any association.

### Strengths and limitations

The main strengths of this study are as follows: *first*, the focus on dietary patterns rather than on single nutrients; *second*, the wide range of possible indicators investigated; *third*, the inclusion of infants with versatile backgrounds obtained by pooling two comparable cohorts and *last*, the contribution of findings obtained from infants in the first year of life, a period for which studies are needed.

The study also comprises some limitations. Including infants only with complete indicator data ensures that one unique data set is used throughout the model reduction. However, results may be biased if missing data do not occur at random. A number of possible indicators differed between completers and non-completers; therefore, the findings from the regression analyses probably represent those families with the largest resources and high health motivation. In addition, the fact that infants in SKOT II are offspring from the TOP study with a focus on maternal diet, physical activity, and breastfeeding-support might influence the associations we report in the present paper. A comparison of SKOT I and SKOT II and possible concerns pooling these data have been reported previously (20). Moreover, it is uncertain if the strength of the association between indicators and dietary patterns are strong enough to pose a biologically relevant difference in health outcomes of the infant. For example, we do not know whether a decrease of 0.22 SD in the Health-Conscious Food pattern per one SD increases in maternal BMI is relevant. In addition, the regression models leave rather large proportions of unexplained variation in the dietary patterns, suggesting that indicators other than those on the long list included here should be investigated, such as parental diet (13). Finally, methodological concerns such as subjective decisions during the PCA analysis and whether or not to adjust for a cohort effect could be posed. The full model of the multiple regression analyses has been carried out, both with and without a cohort variable. The cohort variable was significantly associated with the Family Food and Health-Conscious Food patterns. However, when the cohort variable was included, paternal work situation and maternal BMI did not reach significant levels because these two are the main characteristic differences between the cohorts; therefore, including the cohort variable will blur their association with dietary patterns. To be able to investigate the association between these important indicators and infant dietary patterns, the regression analyses were not adjusted for cohort origin.

## Conclusion

Parental, household, and child characteristics were associated with the dietary patterns Family Food and Health-Conscious Food already at 9 months of age. Our explorative analysis suggests that, for example, families with maternal obesity and multiple children, and perhaps immigrant/descendant families had infants with less favourable dietary patterns. Further analyses of these early indicators of dietary patterns might improve the possibility of identifying infants with an increased risk of developing unfavourable dietary patterns and potentially enable an early targeted preventive support in families with these characteristics.
